# Identification of Breast Cancer Immune Subtypes by Analyzing Bulk Tumor and Single Cell Transcriptomes

**DOI:** 10.3389/fcell.2021.781848

**Published:** 2022-01-03

**Authors:** Jia Yao, Shengwei Li, Xiaosheng Wang

**Affiliations:** ^1^ Department of Breast Surgery, First Affiliated Hospital, College of Medicine, Zhejiang University, Hangzhou, China; ^2^ Biomedical Informatics Research Lab, School of Basic Medicine and Clinical Pharmacy, China Pharmaceutical University, Nanjing, China; ^3^ Cancer Genomics Research Center, School of Basic Medicine and Clinical Pharmacy, China Pharmaceutical University, Nanjing, China; ^4^ Big Data Research Institute, China Pharmaceutical University, Nanjing, China

**Keywords:** breast cancer, clustering analysis, subtyping, transcriptomics, immune signatures, cancer immunotherapy

## Abstract

**Background:** The histological and molecular classification of breast cancer (BC) is being used in the clinical management of this disease. However, subtyping of BC based on the tumor immune microenvironment (TIME) remains insufficiently explored, although such investigation may provide new insights into intratumor heterogeneity in BC and potential clinical implications for BC immunotherapy.

**Methods:** Based on the enrichment scores of 28 immune cell types, we performed clustering analysis of transcriptomic data to identify immune-specific subtypes of BC using six different datasets, including five bulk tumor datasets and one single-cell dataset. We further analyzed the molecular and clinical features of these subtypes.

**Results:** Consistently in the six datasets, we identified three BC subtypes: BC-ImH, BC-ImM, and BC-ImL, which had high, medium, and low immune signature scores, respectively. BC-ImH displayed a significantly better survival prognosis than BC-ImL. Triple-negative BC (TNBC) and human epidermal growth factor receptor-2-positive (HER2+) BC were likely to have the highest proportion in BC-ImH and the lowest proportion in BC-ImL. In contrast, hormone receptor-positive (HR+) BC had the highest proportion in BC-ImL and the lowest proportion in BC-ImH. Furthermore, BC-ImH had the highest tumor mutation burden (TMB) and predicted neoantigens, while BC-ImL had the highest somatic copy number alteration (SCNA) scores. It is consistent with that TMB and SCNA correlate positively and negatively with anti-tumor immune response, respectively. *TP53* had the highest mutation rate in BC-ImH and the lowest mutation rate in BC-ImL, supporting that *TP53* mutations promote anti-tumor immune response in BC. In contrast, *PIK3CA* displayed the highest mutation rate in BC-ImM, while *GATA3* had the highest mutation rate in BC-ImL. Besides immune pathways, many oncogenic pathways were upregulated in BC-ImH, including ErbB, MAPK, VEGF, and Wnt signaling pathways; the activities of these pathways correlated positively with immune signature scores in BC.

**Conclusions:** The tumors with the strong immune response (“hot” tumors) have better clinical outcomes than the tumors with the weak immune response (“cold” tumors) in BC. TNBC and HER2+ BC are more immunogenic, while HR + BC is less immunogenic. Certain HER2+ or HR + BC patients could be propitious to immunotherapy in addition to TNBC.

## Background

Breast cancer (BC) is the most common cancer and the second leading cause of cancer death in women (Siegel et al., 2020). Abundant evidence has shown that BC is highly heterogeneous in molecular profiles (Yersal and Barutca, 2014). For example, based on differential expression of 50 genes (PAM50), BC is classified into five subtypes: basal-like, HER2-enriched, luminal A, luminal B, and normal-like (Picornell et al., 2019). Based on the expression of estrogen receptor (ER), progesterone receptor (PR), or human epidermal growth factor receptor-2 (HER2), BC can be divided into ER+ and ER-, PR+ and PR-, or HER2+ and HER2- (Fragomeni et al., 2018). The main advantage of BC subtyping is its advising optimal treatments (Yang and Polley, 2019). Traditional therapeutic strategies for BC included surgery, radiotherapy, chemotherapy, and targeted therapy. In particular, targeted therapies for ER + or HER2+ BC have achieved great successes (Yang and Polley, 2019). However, some metastatic or refractory BCs, such as brain metastatic HER2+ BCs, have limited treatment options. In addition, some aggressive BC subtypes, such as triple-negative BCs (TNBCs) which constitute 15–20% of BCs, have no effective targeted therapies.

Immunotherapies, such as immune checkpoint inhibitors (ICIs) (Del Paggio, 2018), have exhibited successes in treating various cancers, including TNBC. Nevertheless, currently, only a subset of cancer patients can benefit from such therapies (Braun et al., 2016). To identify the subset of cancer patients responsive to immunotherapies, certain biomarkers have been discovered, including PD-L1 expression (Davis and Patel, 2019), mismatch repair deficiency (Le et al., 2015), and high tumor mutation burden (TMB) (Goodman et al., 2017). Besides, the tumor immune microenvironment (TIME) plays a crucial role in immunotherapeutic response (Gajewski, 2015). In general, “hot” tumors with a high level of T cell infiltration tends to respond better to immunotherapies than “cold” tumors with sparse T cell infiltration (Gajewski, 2015). Therefore, distinguishing between “hot” and “cold” tumors may identify cancer patients responsive to immunotherapies. In a previous study (He et al., 2018), we proposed an unsupervised machine learning method to identify “hot” and “cold” TNBC based on immunogenomic profiling. In this study, to characterize the immunological landscape of BC, not limited to TNBC, we identified immune-specific subtypes of BC by unsupervised clustering analysis of five transcriptomic datasets. We comprehensively characterized the molecular and clinical features of these subtypes. Furthermore, we compared the immune-specific subtyping with the traditional molecular subtyping systems of BC. Our data may provide new insights into associations of BC immunity with its molecular and clinical features and subtypes, as well as potential clinical implications for BC immunotherapies.

## Methods

### Datasets

We downloaded The Cancer Genome Atlas Breast Invasive Carcinoma (TCGA-BRCA) dataset, including RNA-Seq gene expression profiles, somatic mutation profiles, protein expression profiles, and clinical data, from the genomic data commons data portal (https://portal.gdc.cancer.gov/). The METABRIC BC dataset, including gene expression profiles, somatic mutation profiles, and clinical data, were downloaded from cBioPortal (http://www.cbioportal.org). We obtained other three BC transcriptomic datasets (GSE24450, GSE 2034, and GSE11121) from the NCBI gene expression omnibus (GEO) (https://www.ncbi.nlm.nih.gov/geo/). In addition, we downloaded a single-cell RNA sequencing (scRNA-seq) dataset (GSE75688 (Chung et al., 2017)) for BC from the NCBI GEO. A summary of these datasets is provided in Supplementary Table S1.

### Calculation of Immune Signatures or Pathways’ Enrichment Scores

We calculated the enrichment score of an immune signature or pathway in a tumor sample by the single-sample gene-set enrichment analysis (ssGSEA) (Hänzelmann et al., 2013) of the expression profiles of its marker or pathway gene set. The ssGSEA is an extension of the GSEA method, which outputs the enrichment scores of the input gene sets in different samples by inputting an expression matrix and a list of gene sets. The marker or pathway genes of immune signatures or pathways are shown in Supplementary Table S2.

### Identification of BC Subtypes

Based on the enrichment scores of 28 immune cell types (Charoentong et al., 2017), we identified BC subtypes by hierarchical clustering. The hierarchical clustering is an unsupervised machine learning algorithm that determines the similarity between data points in each category by calculating the distance between them and all data points; a smaller distance indicates a higher similarity, and combining two data points or categories with the closest distance generates a clustering tree. The 28 immune cell types included CD56-bright natural killer (NK) cells, effector memory CD4 T cells, eosinophil, CD56-dim NK cells, type 17 T helper cells, activated B cells, monocytes, memory B cells, activated CD4 T cells, type 2 T helper cells, plasmacytoid dendritic cells, neutrophils, macrophages, effector memory CD8 T cells, myeloid-derived suppressor cell (MDSC), immature B cells, T follicular helper cells, NK cells, immature dendritic cells, mast cells, type 1 T helper cells, activated dendritic cells, central memory CD4 T cells, gamma delta T cells, central memory CD8 T cells, regulatory T cells, activated CD8 T cells, and natural killer T cells (Charoentong et al., 2017).

### Survival Analysis

We compared overall survival (OS) and disease-free survival (DFS) rates among BC subtypes using the Kaplan-Meier (K-M) method (Bland and Altman, 1998). K-M curves were utilized to display the survival rate differences, whose significances were evaluated by log-rank tests.

### TMB and SCNA Score in Tumors

A tumor’s TMB was defined as its total count of somatic mutations, and a tumor’s SCNA score was the sum of its recurrent SCNAs (Taylor et al., 2018).

### Immune Scores of Tumors

The immune score of a tumor reflects its immune infiltration level, which was calculated by ESTIMATE (Yoshihara et al., 2013) with the input of the expression profiles of immune genes.

### Logistic Regression Analysis

We compared the contribution of TMB and SCNA score in predicting high-immune-signature-score (>median) versus low-immune-signature-score (<median) BC using the logistic regression analysis. In the logistic regression analysis, the R function “glm” was utilized to fit the binary model, and the R function “lm.beta” in the R package “QuantPsyc” was used to calculate the standardized regression coefficients (β values).

### Pathway Analysis

We first identified differentially expressed genes (DEGs) between BC-ImH and BC-ImL using two-tailed Student’s *t* test with a threshold of adjusted *p*-value < 0.05 and fold change of mean expression levels >1.5. By input of the upregulated DEGs in a BC subtype into GSEA (Liu et al., 2018), we obtained the KEGG (Liu et al., 2019) pathways upregulated in the subtype with a threshold of adjusted *p*-value < 0.05.

### Statistical Analysis

In class comparison, if the data were non-normally distributed, we used the Mann–Whitney *U* test, otherwise, we used Student’s *t* test. We used the Spearman method to calculate correlations between immune scores and pathways’ enrichment scores. In the evaluation of associations between two categorical variables, we utilized Fisher’s exact test. To correct *p* values in multiple tests, we calculated the false discovery rate (FDR) by the Benjamini-Hochberg method (Benjamini and Hochberg, 1995). We performed all statistical analyses in the R programming environment (version 3.6.0).

## Results

### Identification of Immune Subtypes of BC

**FIGURE 1:**
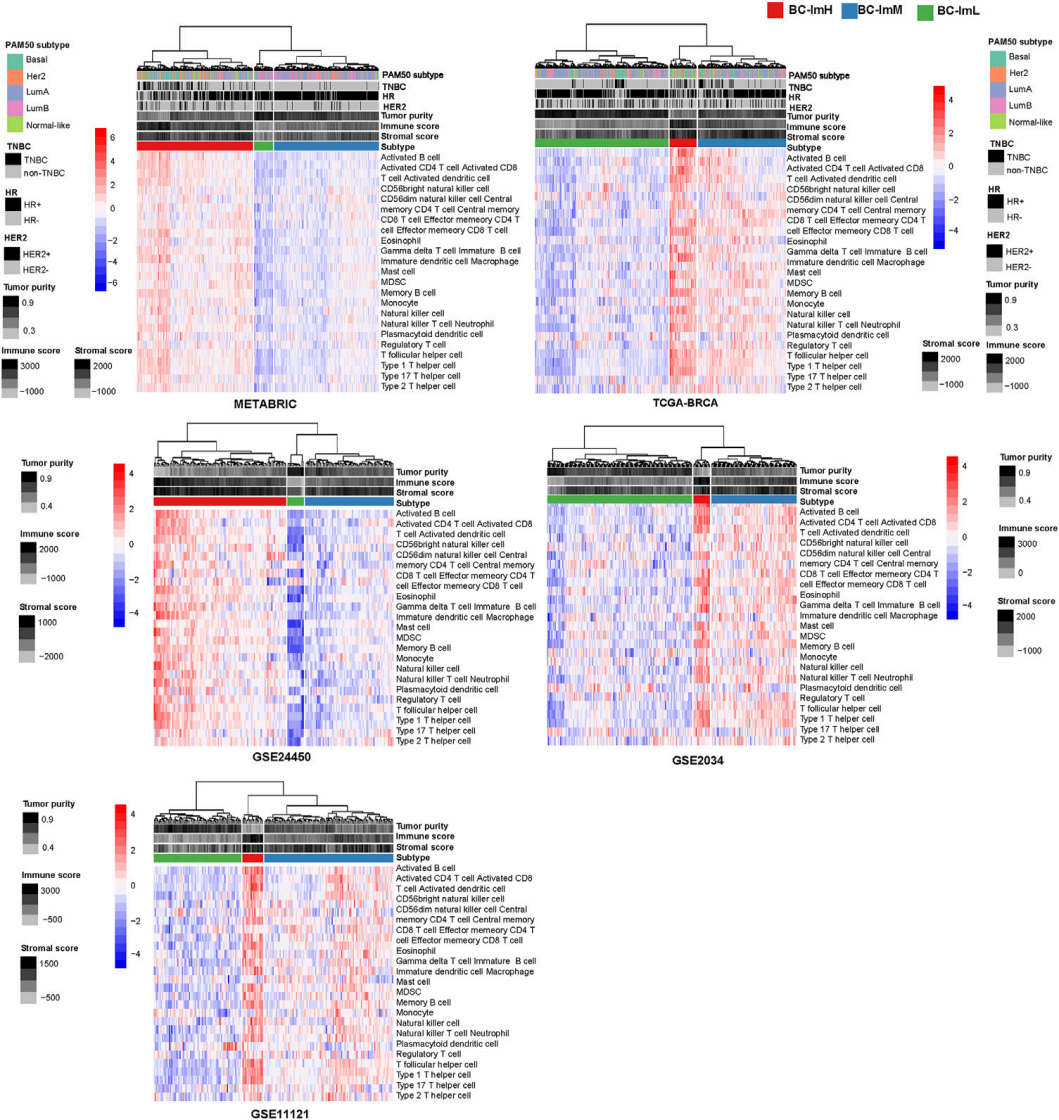
Hierarchical clustering of breast cancer (BC) in five transcriptomic datasets based on the enrichment scores of 28 immune cell types. The clustering analysis identifying three immune subtypes of BC: BC-ImH, BC-ImM, and BC-ImL, with high, medium, and low immune signature scores, respectively, consistently in the five datasets.

**FIGURE 2:**
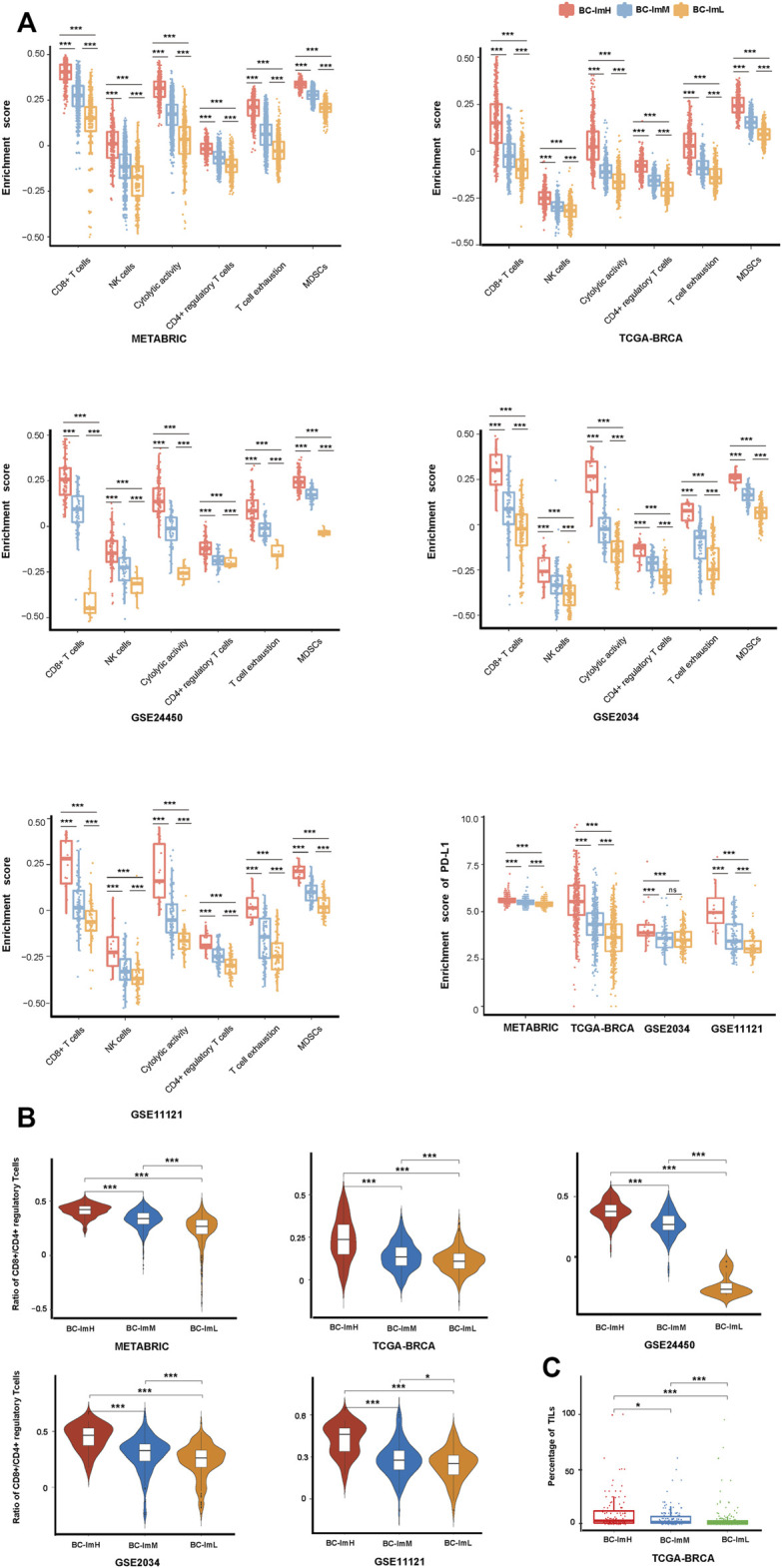
Comparisons of the enrichment scores of immune signatures among the three BC subtypes. Comparisons of the enrichment scores of immunostimulatory signatures (NK cells, CD8^+^ T cells, and immune cytolytic activity) and immunosuppressive signatures (CD4^+^ regulatory T cells, myeloid-derived suppressor cells (MDSCs), T cell exhaustion, and PD-L1) **(A)**, ratios of immunostimulatory over immunosuppressive signatures (CD8+/CD4+ regulatory T cells) **(B)**, and the percentage of tumor infiltrating lymphocytes (TILs) **(C)** among the three BC subtypes. The one-tailed Mann–Whitney *U* test or two-tailed Student’s *t* test *p* values are shown. *p < 0.05, **p < 0.01, ***p < 0.001, ^ns^ *p* ≥ 0.05. It also applies to the following figures.

Using hierarchical clustering, we identified immune subtypes of BC based on the enrichment scores of 28 immune cell types (Charoentong et al., 2017). We performed the clustering analysis in five BC transcriptomic datasets (TCGA-BRCA, METABRIC, GSE24450, GSE 2034, and GSE11121), respectively. Consistently in these datasets, we identified three immune subtypes of BC, termed BC-ImH, BC-ImM, and BC-ImL, which had high, medium, and low immune signature scores, respectively (Figure 1). We further compared the enrichment scores of both immunostimulatory signatures (NK cells, CD8^+^ T cells, and immune cytolytic activity) and immunosuppressive signatures (CD4^+^ regulatory T cells, myeloid-derived suppressor cells (MDSCs), and T cell exhaustion) among the three subtypes. Interestingly, all these immune signatures showed the highest enrichment scores in BC-ImH and the lowest enrichment scores in BC-ImL (one-tailed Mann–Whitney *U* test, *p* < 0.001) in the five datasets (Figure 2A). In addition, PD-L1, also an immunosuppressive signature, was likely to have the highest and lowest mRNA expression levels in BC-ImH and BC-ImL, respectively (two-tailed Student’s *t* test, *p* < 0.05) (Figure 2A). Meanwhile, the ratios of immunostimulatory over immunosuppressive signatures (CD8+/CD4+ regulatory T cells), which were the base-2 log-transformed values of the geometric mean expression levels of all marker genes of CD8^+^ T cells divided by those of CD4^+^ regulatory T cells, were the highest in BC-ImH and the lowest in BC-ImL (one-tailed Mann–Whitney *U* test, *p* < 0.05) in these datasets (Figure 2B). Furthermore, we compared the percentage of tumor infiltrating lymphocytes (TILs) among the subtypes based on the pathology slide data in TCGA-BRCA. As expected, the percentage of TILs followed the pattern: BC-ImH > BC-ImM > BC-ImL (*p* < 0.05) (Figure 2C). Altogether, these results supported that BC-ImH and BC-ImL had the strongest and weakest anti-tumor immune response, respectively.

### Clinical Features of the BC Subtypes

**FIGURE 3:**
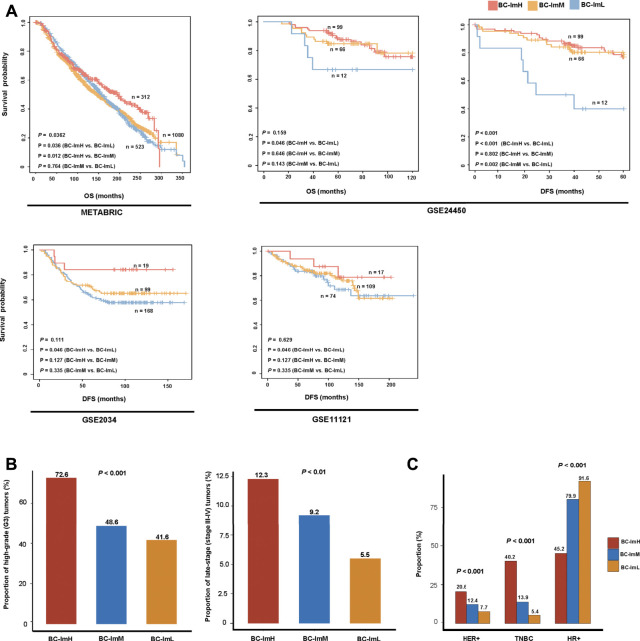
Comparisons of clinical features among the BC subtypes. **(A)** Comparisons of overall survival (OS) and disease-free survival (DFS) time among the BC subtypes by Kaplan–Meier curves. The log-rank test *p* values are shown. Comparisons of the proportion of high-grade (G3) tumors, the proportion of late-stage (stage III–IV) tumors **(B)**, and proportions of HER2^+^, TNBC, HR^+^ tumors **(C)** among the BC subtypes in METABRIC. The Fisher’s exact test *p* values are shown.

We compared survival prognosis among the three subtypes in the five datasets. Interestingly, in four datasets (METABRIC, GSE24450, GSE 2034, and GSE11121), BC-ImH displayed significantly better OS and/or DFS than BC-ImL (log-rank test, *p* < 0.05) (Figure 3A). In addition, in METABRIC, BC-ImH showed significantly better OS than BC-ImM (*p* = 0.012), and in GSE24450, BC-ImM had significantly better DFS than BC-ImL (*p* = 0.002) (Figure 3A). These results imply a positive association between immune infiltration levels and survival prognosis in BC.

Tumor grade indicates how abnormal the tumor cells look under a microscope compared to normal cells and how fast a tumor is likely to grow and spread. In METABRIC, BC-ImH and BC-ImL harbored the largest and smallest proportion of high-grade (G3) tumors, respectively (BC-ImH (72.6%) versus BC-ImM (48.6%) versus BC-ImL (41.6%)) (Fisher’s exact test, *p* < 0.001), and BC-ImH and BC-ImL had the largest and smallest proportion of late-stage (stage III-IV) tumors, respectively (BC-ImH (12.3%) versus BC-ImM (9.2%) versus BC-ImL (5.5%)) (*p* < 0.01) (Figure 3B). These results suggest that anti-tumor immune signatures increase with tumor progression in BC.

In METABRIC, HER2+ tumors had the highest proportion in BC-ImH and the lowest proportion in BC-ImL (BC-ImH (20.6%) versus BC-ImM (12.4%) versus BC-ImL (7.7%)) (*p* < 0.001) (Figure 3C). TNBC also had the highest proportion in BC-ImH and the lowest proportion in BC-ImL (BC-ImH (40.2%) versus BC-ImM (13.9%) versus BC-ImL (5.4%)) (*p* < 0.001) (Figure 3C). In contrast, tumors with hormone receptor-positive (HR+), namely ER+ and/or PR+, had the highest proportion in BC-ImL and the lowest proportion in BC-ImH (BC-ImH (45.2%) versus BC-ImM (79.9%) versus BC-ImL (91.6%)) (*p* < 0.001) (Figure 3C). These results indicate that TNBC and HER2+ tumors are more immunogenic, while HR + tumors are less immunogenic. This is in accordance with previous reports (Safonov et al., 2017; Liu et al., 2018; Liu et al., 2019).

### Genomic Features of the BC Subtypes

**FIGURE 4:**
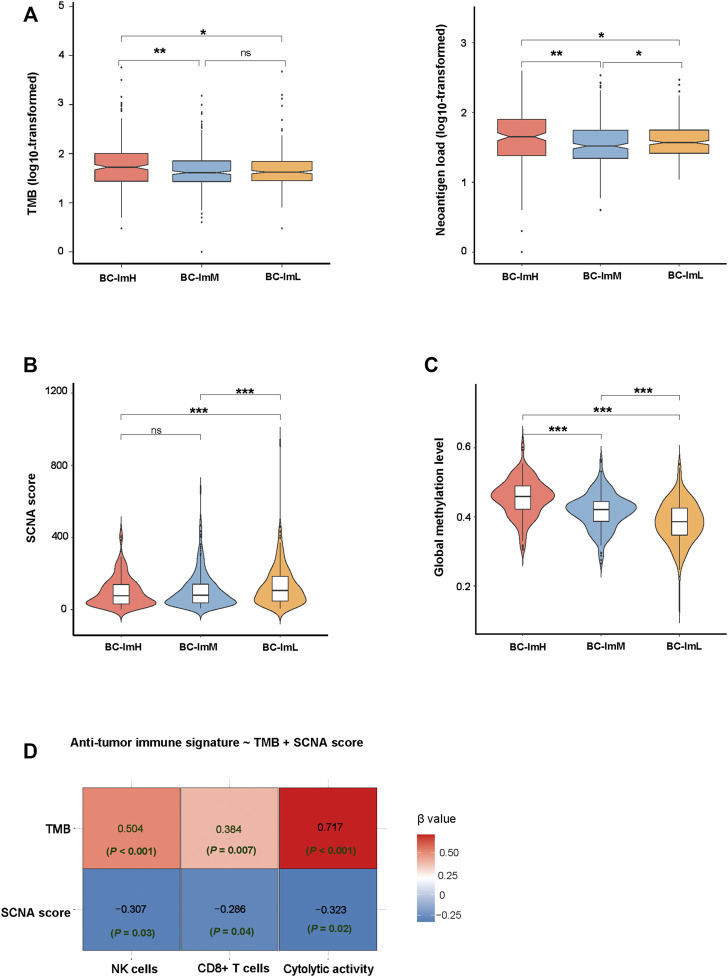
Comparisons of genomic features among the BC subtypes in TCGA-BRCA. Comparisons of TMB and neoantigen load **(A)**, SCNA scores **(B)**, and global methylation levels **(C)** among the BC subtypes. The onetailed Mann–Whitney *U* test p values are shown in **(A–C)**. **(D)** Prediction of the scores (high (>median) versus low (<median)) of three immune signatures (NK cells, CD8^+^ T cells, and immune cytolytic activity) using TMB and SCNA score by the logistic regression model. TMB, tumor mutation burden; SCNA, somatic copy number alteration.

Somatic mutations and copy number alterations (SCNAs) are common genomic features in tumors, which are associated with anti-tumor immune response (Davoli et al., 2017). We found that BC-ImH had significantly higher TMB than BC-ImM and BC-ImL (one-tailed Mann–Whitney *U* test, *p* < 0.05) in TCGA-BRCA (Figure 4A). Accordingly, BC-ImH involved more abundant neoantigens (Rooney et al., 2015) than BC-ImM and BC-ImL (Figure 4A). In contrast, BC-ImL had significantly higher SCNA scores than BC-ImH and BC-ImM (*p* < 0.001) (Figure 4B). These results conform to previous findings that TMB and SCNAs correlate positively and negatively with anti-tumor immune response, respectively. Previous studies suggest that reduced DNA methylation levels can promote tumor immune evasion (Jung et al., 2019). Consistent with the suggestion, the global methylation levels (Jung et al., 2019) were the highest in BC-ImH and the lowest in BC-ImL in TCGA-BRCA (*p* < 0.001) (Figure 4C).

In predicting the scores (high (>median) versus low (<median)) of three immune signatures (NK cells, CD8^+^ T cells, and immune cytolytic activity) using TMB and SCNA score by the logistic regression analysis, TMB was a significant and positive predictor, while SCNA score was a significant and negative predictor (*p* < 0.05) (Figure 4D). Again, this is consistent with that TMB and SCNAs have a positive and negative correlation with anti-tumor immune response, respectively. Interestingly, TMB displayed greater contributions in predicting the three immune signatures than the SCNA score, as evidenced by its smaller *p* values and larger absolute β values in the logistic regression models.

### Pathways Upregulated in the BC Subtypes

**FIGURE 5:**
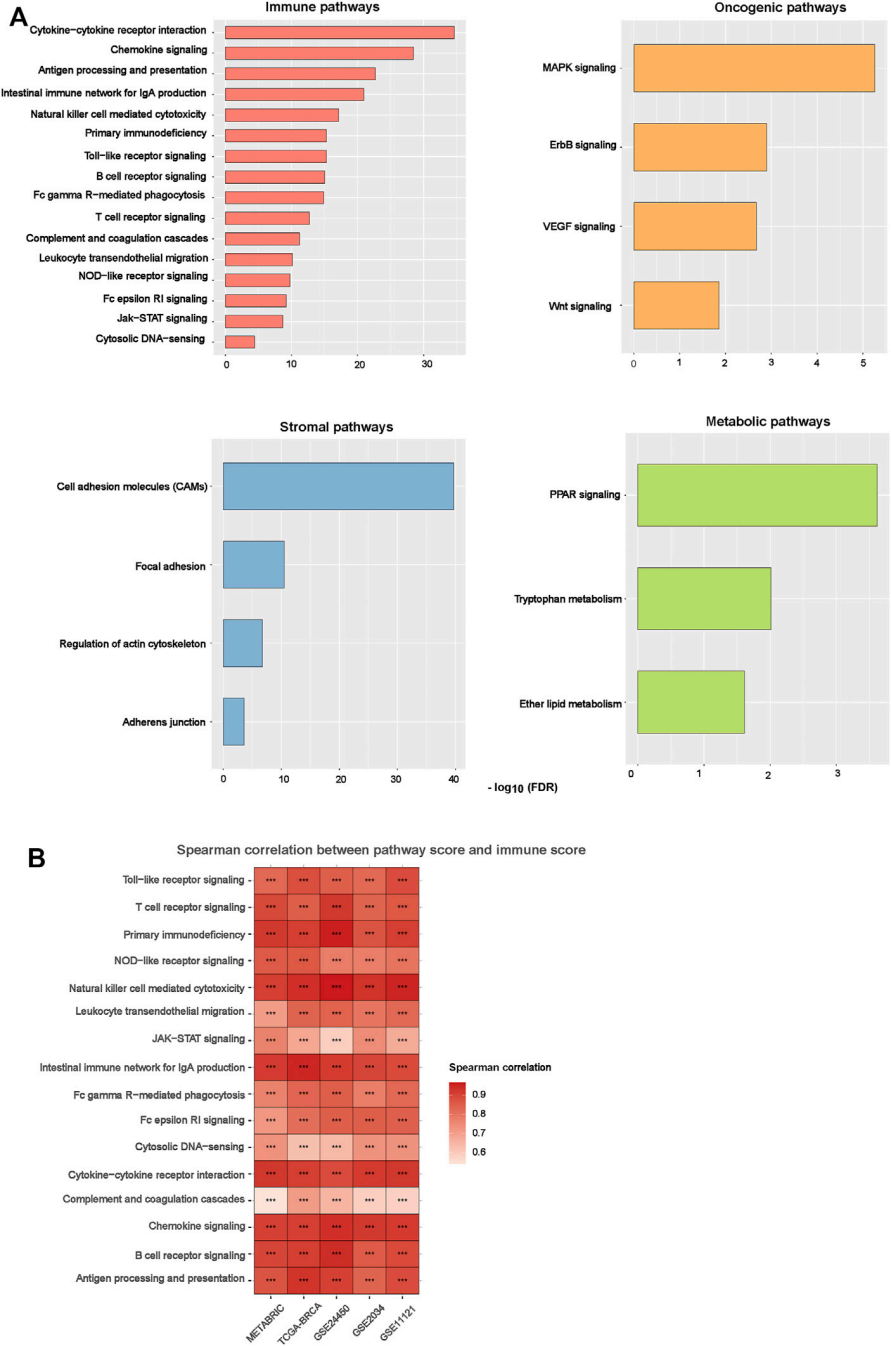
Pathways upregulated in the BC subtypes. **(A)** The KEGG pathways upregulated in BC-ImH versus BC-ImL identified in the five BC datasets in common. **(B)** Spearman correlations between the enrichment scores of pathways upregulated in BC-ImH and immune scores in the five BC datasets. The immune score of a tumor represents its immune infiltration level, which was calculated by ESTIMATE (Yoshihara et al., 2013).

Based on DEGs between BC-ImH and BC-ImL, we identified KEGG pathways upregulated in BC-ImH and BC-ImL using the GSEA tool (Subramanian et al., 2005). The pathways upregulated in BC-ImH and BC-ImL were identified based on significantly upregulated DEGs in BC-ImH and BC-ImL, respectively, using a threshold of adjusted *p*-value < 0.05. We performed the pathway analysis in each of the five datasets and found 54 and 0 pathways upregulated in BC-ImH and BC-ImL, respectively, consistent in the five datasets. The pathways upregulated in BC-ImH were mainly involved in immune, stromal, oncogenic, and metabolic processes (Figure 5A). The immune pathways included antigen processing and presentation, B cell receptor signaling, chemokine signaling, complement, and coagulation cascades, cytokine-cytokine receptor interactions, cytosolic DNA-sensing, Fc epsilon RI signaling, Fc gamma R-mediated phagocytosis, intestinal immune network for IgA production, Jak-STAT signaling, leukocyte transendothelial migration, natural killer cell-mediated cytotoxicity, NOD-like receptor signaling, primary immunodeficiency, T cell receptor signaling, and Toll-like receptor signaling. The stromal signature pathways included adherens junction, cell adhesion molecules, focal adhesion, and regulation of actin cytoskeleton. The oncogenic pathways included ErbB signaling, MAPK signaling, VEGF signaling, and Wnt signaling. The metabolic pathways included ether lipid metabolism, PPAR signaling, and tryptophan metabolism. The upregulation of various immune pathways in BC-ImH is consistent with the strongest immune signatures in this subtype. Furthermore, we found that the enrichment scores of these pathways (except the immune pathways) upregulated in BC-ImH were likely to correlate positively with immune scores in these datasets (Spearman correlation, *p* < 0.05) (Figure 5B). Again, these results conformed to the fact that BC-ImH was most enriched with immune signatures.

### Somatic Mutation Profiles in the BC Subtypes

**FIGURE 6:**
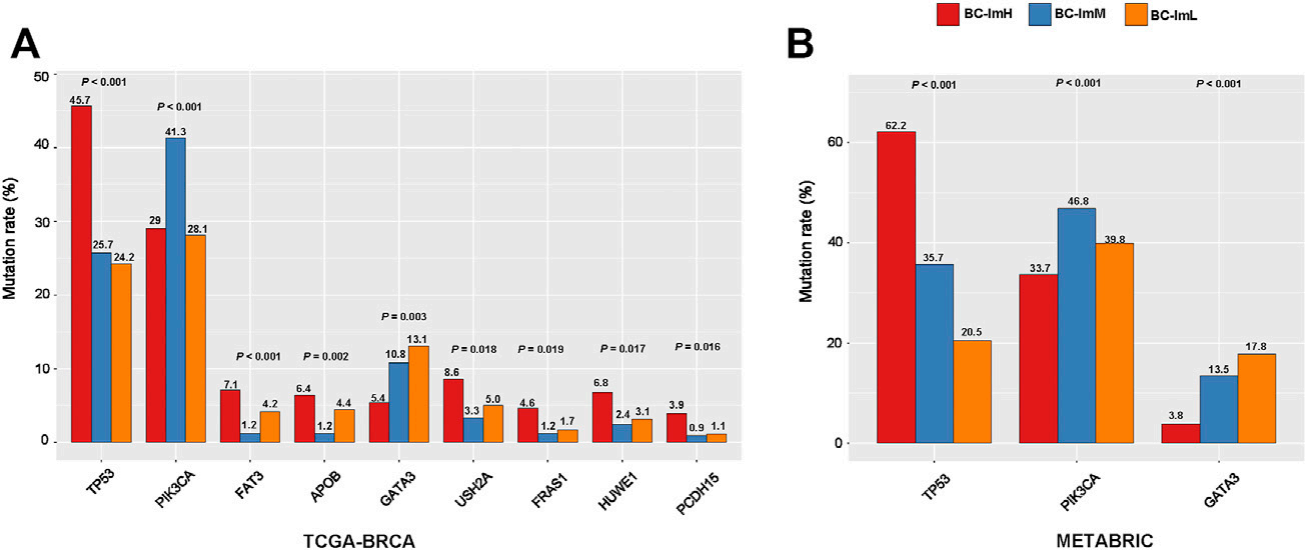
Comparisons of somatic mutation profiles among the BC subtypes. **(A)** nine genes showing significantly different mutation frequencies among the BC subtypes in TCGA-BRCA. **(B)** three genes show significantly different mutation frequencies among the BC subtypes in METABRIC. The Fisher’s exact test *p* values are shown.

We compared somatic mutation profiles among the BC subtypes in TCGA-BRCA, which involved whole exome sequencing data. We found nine genes having significantly different mutation frequencies among the BC subtypes (Fisher’s exact test, FDR <0.15) (Figure 6A). These genes included *TP53*, *PIK3CA*, *FAT3*, *APOB*, *GATA3*, *USH2A*, *FRAS1*, *HUWE1*, and *PCDH15*. Notably, *TP53* had the highest mutation rate in BC-ImH and the lowest mutation rate in BC-ImL (BC-ImH (45.7%) versus BC-ImM (25.7%) versus BC-ImL (24.2%)). This is in line with our previous finding that *TP53* mutations promote anti-tumor immune response in BC (Liu et al., 2019). Also, *FAT3*, *APOB*, *USH2A*, *FRAS1*, *HUWE1*, and *PCDH15* showed the highest mutation rate in BC-ImH. In contrast, *PIK3CA* displayed the highest mutation rate in BC-ImM (BC-ImH (28.2%) versus BC-ImM (41.3%) versus BC-ImL (28.1%)), while *GATA3* had the highest mutation rate in BC-ImL (BC-ImH (5.4%) versus BC-ImM (10.8%) versus BC-ImL (13.1%)). It is justified that *GATA3* showed the highest mutation rate in BC-ImL since GATA3 plays an important role in the regulation of innate and adaptive immunity (Tindemans et al., 2014). We further verified that the mutation rates of *TP53*, *PIK3CA*, and *GATA3* followed the patterns of BC-ImH > BC-ImM > BC-ImL, BC-ImM > BC-ImL > BC-ImH, and BC-ImH < BC-ImM < BC-ImL, respectively, in METABRIC, which involved targeted exome sequencing data (Figure 6B).

### Protein Expression Profiles in the BC Subtypes

**FIGURE 7:**
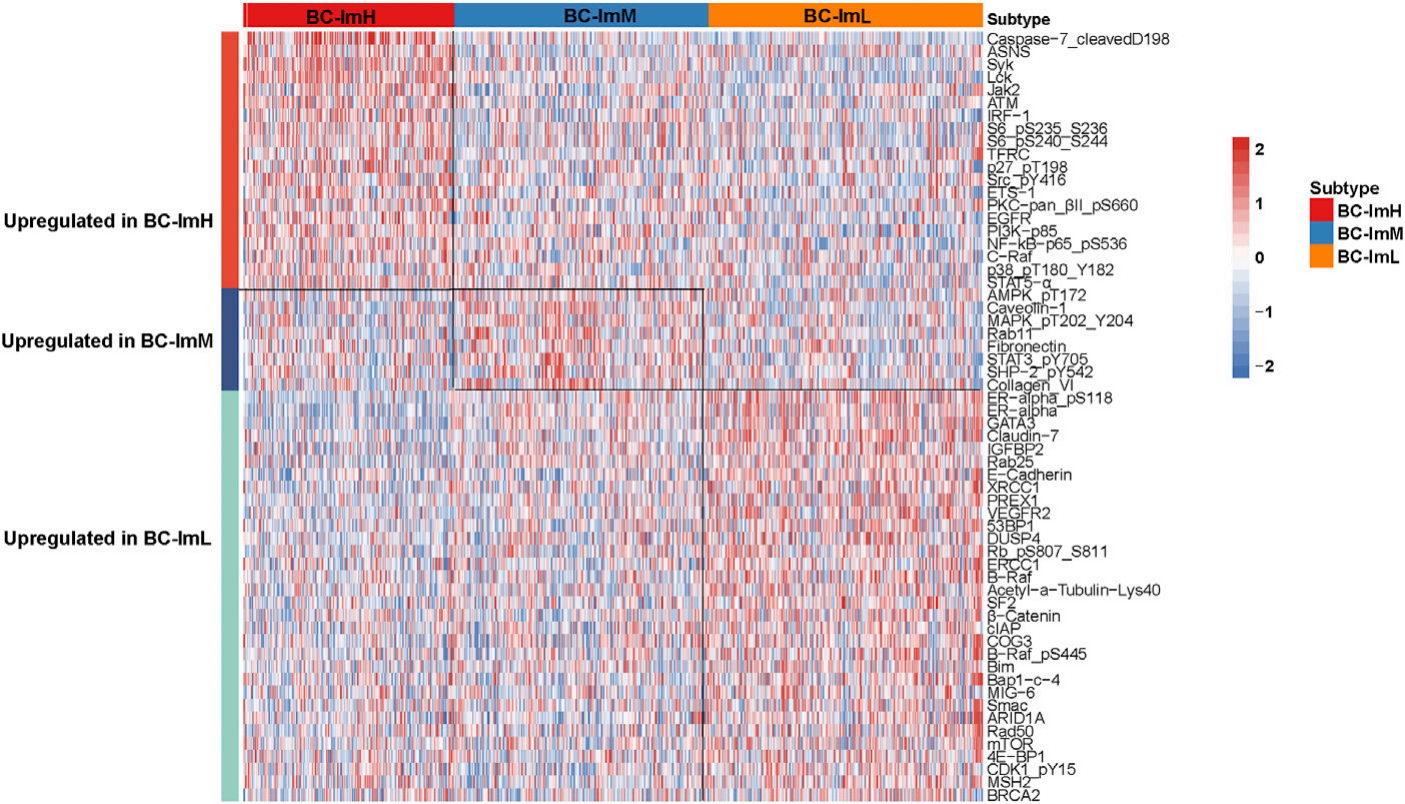
Heatmap showing differentially expressed proteins among the BC subtypes in TCGA-BRCA.

We compared the expression levels of 261 proteins among the BC subtypes in TCGA-BRCA. We found 20 proteins showing significantly higher expression levels in BC-ImH than in both BC-ImM and BC-ImL (two-tailed Student’s *t* test, FDR <0.05) (Figure 7). These proteins included Caspase-7_cleavedD198, ASNS, Syk, Lck, Jak2, ATM, IRF-1, S6_pS235_S236, S6_pS240_S244, TFRC, p27_pT198, Src_pY416, ETS-1, PKC-pan_βII_pS660, EGFR, PI3K-p85, NF-kB-p65_pS536, C-Raf, p38_pT180_Y182, and STAT5-α. In fact, many of these proteins have been shown to have a positive correlation with immune response in cancer, such as Caspase-7 (Samstein et al., 2019), Jak2 (Conze et al., 2002), NF-kB (Taniguchi and Karin, 2018), and STAT5 (Ding et al., 2020). In contrast, 32 proteins had significantly higher expression levels in BC-ImL than in both BC-ImH and BC-ImM (Figure 7). Many of these proteins have been shown to have a negative correlation with immune response in cancer. For example, our previous study revealed that ER-α inhibited anti-tumor immune response in BC (Liu et al., 2019). BRAF inhibition may promote anti-tumor immune response (Ilieva et al., 2014). CDK1 is a regulator of cell cycle, whose activation inhibits anti-tumor immune response (Goel et al., 2017). In addition, we found eight proteins having significantly higher expression levels in BC-ImM than in both BC-ImH and BC-ImL (Figure 7). These proteins included AMPK_pT172, Caveolin-1, MAPK_pT202_Y204, Rab11, Fibronectin, STAT3_pY705, SHP-2_pY542, and Collagen_VI.

### Immunological Classification of BC Single Cells

**FIGURE 8:**
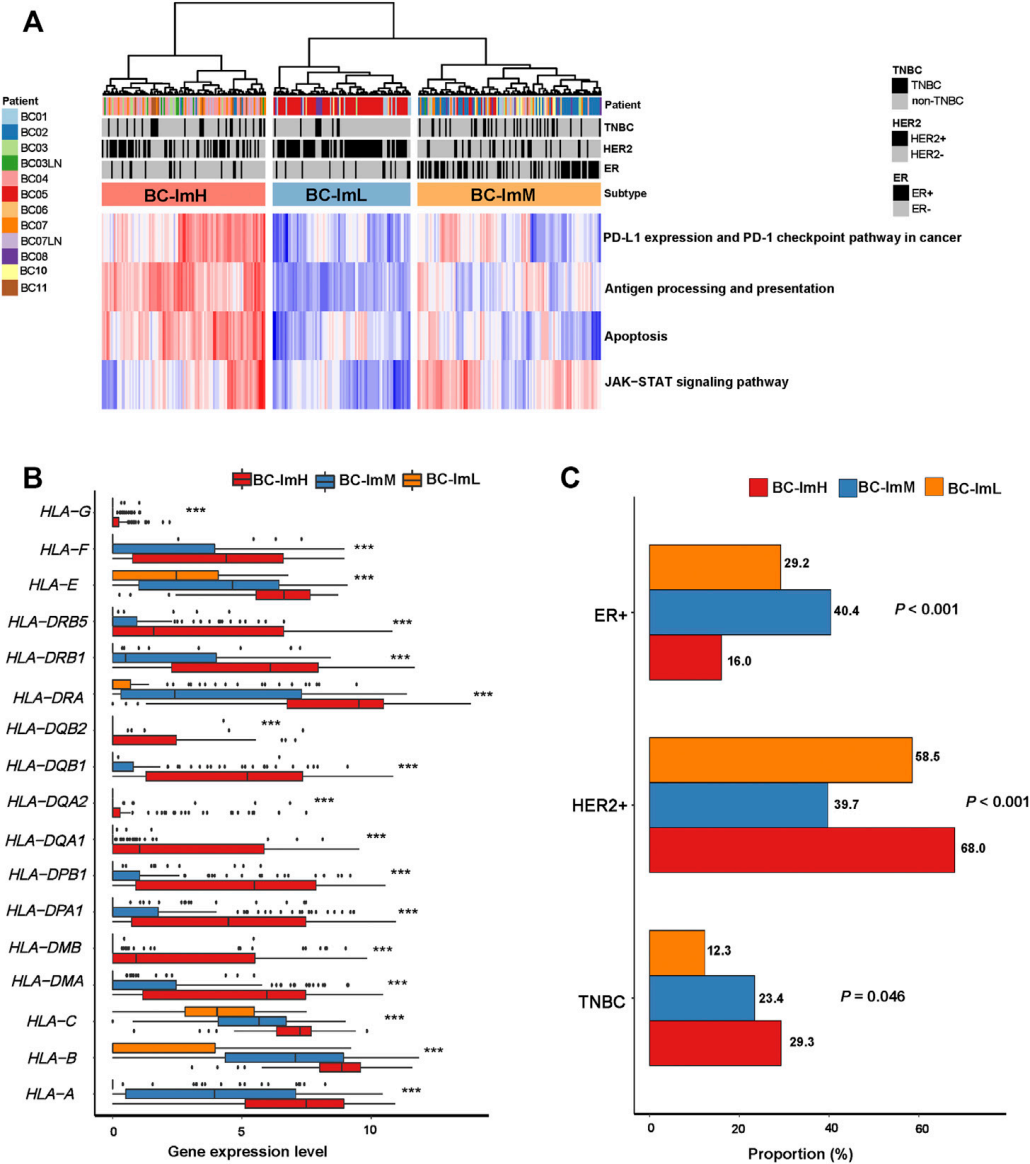
Validation of the BC subtyping method in a single-cell RNA-seq dataset. **(A)** Hierarchical clustering of 317 tumor cells from ten BC patients based on the enrichment scores of four immune-related pathways. **(B)** Comparisons of the expression levels of 19 human leukocyte antigen (HLA) genes among the subtypes. One-way analysis of variance (ANOVA) test *p* values are shown. **(C)** Comparisons of proportions of TNBC, HER2+, and ER + tumor cells among the subtypes. The Fisher’s exact test *p* values are shown.

We used the immune signature scores-based clustering method to analyze a scRNA-seq dataset (GSE75688 (Chung et al., 2017)). This dataset involved gene expression profiles in 317 tumor cells from ten BC patients. We hierarchically clustered these tumor cells based on their expression levels (enrichment scores) of four immune pathways, including antigen processing and presentation, PD-L1 expression and PD-1 checkpoint pathway in cancer, JAK-STAT signaling, and apoptosis. We used the four immune signatures instead of the 28 immune cell types in analyzing the scRNA-seq dataset because these immune signatures are expressed in tumor cells themselves. Likewise, we classified the 317 tumor cells into three subgroups: BC-ImH, BC-ImM, and BC-ImL (Figure 8A). We compared the expression levels of 19 human leukocyte antigen (HLA) genes among these subtypes. Strikingly, almost all the HLA genes showed the expression pattern: of BC-ImH > BC-ImM > BC-ImL (two-tailed Student’s *t* test, *p* < 0.02) (Figure 8B). These results confirmed that BC-ImH and BC-ImL had the highest and lowest immunity among the subtypes.

Likewise, in the scRNA-seq dataset, single cells from TNBC had the highest proportion in BC-ImH and the lowest proportion in BC-ImL (BC-ImH (29.3%) versus BC-ImM (23.4%) versus BC-ImL (12.3%)) (*p* = 0.046) (Figure 8C). Single cells from HER2+ BC had the highest proportion in BC-ImH and the lowest proportion in BC-ImM (BC-ImH (68.0%) versus BC-ImM (39.7%) versus BC-ImL (58.5%)) (*p* < 0.001) (Figure 8C). Single cells from ER + BC had the highest proportion in BC-ImM and the lowest proportion in BC-ImH (BC-ImH (16.0%) versus BC-ImM (40.4%) versus BC-ImL (29.2%)) (*p* < 0.001) (Figure 8C). Overall, these results confirmed that TNBC and HER2+ tumor cells are more immunogenic, while HR + tumor cells are less immunogenic.

## Discussion

We performed an immunological classification of BC based on bulk and single cell transcriptomes. We identified three BC subtypes: BC-ImH, BC-ImM, and BC-ImL, which showed high, medium, and low immune signature scores, respectively (Figure 1). We demonstrated that this classification method was producible and stable by analyzing six different datasets, including five bulk tumor datasets and one single cell dataset. Our results support that the tumors with the strong immune response (“hot” tumors) have better clinical outcomes than the tumors with the weak immune response (“cold” tumors), as was also observed in many other cancer types, including head and neck squamous cell cancer (Lyu et al., 2019) and gastric cancer (Jiang et al., 2018). It should be noted that the positive association between the enrichment levels of TILs and clinical outcomes is not necessarily valid in all cancer types. In fact, in certain cancer types, such as prostate cancer (Thorsson et al., 2018) and gliomas (Pombo Antunes et al., 2020), the tumors with strong immune response often have worse clinical outcomes than the tumors with weak immune response. Thus, the association between immune response and clinical outcomes depends on the tissue or cellular origins of cancers. The main mechanism underlying this difference could be that the immune response is the tumor progression-promoting inflammation or immune cell-mediated killing of tumor cells.

TMB and SCNAs have a positive and negative correlation with immune response in cancer, respectively (Davoli et al., 2017). Consistent with this observation, BC-ImH had the highest TMB, while BC-ImL had the highest SCNA scores (Figures 4A,B). The logistic regression analysis showed that TMB contributed to the alterations of immune activity more strongly than SCNAs (Figure 4D). Because high TMB is likely to generate more neoantigens, it is justified that BC-ImH has the strongest anti-tumor immune response. Interestingly, PD-L1, an immunosuppressive signature, and biomarker for cancer immunotherapy, was more highly expressed in BC-ImH and more lowly expressed in BC-ImL. Because high TMB (Goodman et al., 2017), PD-L1 expression (Patel and Kurzrock, 2015), and high level of TILs (Haanen, 2017) are predictors of a favorable response to ICIs, PC-ImH would respond best to ICIs among the subtypes. This is supported by that TNBC, a BC subtype with the highest proportion in BC-ImH, is the BC subtype propitious to be treated by ICIs in clinical practice (Emens, 2021). Nevertheless, our data showed that around 60% of TNBC patients were not classified into BC-ImH, suggesting that many TNBC patients may not have an active response to ICIs. On the other hand, many HER2+ or HR + BC patients belonged to BC-ImH, suggesting that a certain proportion of HER2+ or HR + patients could respond well to ICIs. Thus, for the HER2+ or HR + patients of BC-ImH, a combination of targeted therapy and immunotherapy could be a viable option.

## Conclusion

BC can be classified into three subtypes based on immune signature scores. The tumors with the strong immune response (“hot” tumors) have better clinical outcomes than the tumors with the weak immune response (“cold” tumors) in BC. TNBC and HER2+ BC are more immunogenic, while HR + BC is less immunogenic. Certain HER2+ or HR + BC patients could be propitious to immunotherapy in addition to TNBC.

## Data Availability

The original contributions presented in the study are included in the article/Supplementary Material, further inquiries can be directed to the corresponding author.

## Abbreviations

BC, Breast cancer; TIME, tumor immune microenvironment; TNBC, triple-negative BC; HER2+, human epidermal growth factor receptor-2-positive; HR+, hormone receptor-positive; TMB, tumor mutation burden; SCNA, somatic copy number alteration; BC, Breast cancer; ER, estrogen receptor; PR, progesterone receptor; ICIs, immune checkpoint inhibitors; TCGA-BRCA, The Cancer Genome Atlas Breast Invasive Carcinoma; GEO, gene expression omnibus; scRNA-seq, single-cell RNA sequencing; ssGSEA, single-sample gene-set enrichment analysis; MDSC, myeloid-derived suppressor cell; OS, overall survival; DFS, disease-free survival; K-M, Kaplan-Meier; DEGs, differentially expressed genes; FDR, false discovery rate.
