# Corrigendum: Identification of breast cancer immune subtypes by analyzing bulk tumor and single cell transcriptomes

**DOI:** 10.3389/fcell.2022.948644

**Published:** 2022-08-26

**Authors:** Jia Yao, Shengwei Li, Xiaosheng Wang

**Affiliations:** ^1^ Department of Breast Surgery, First Affiliated Hospital, College of Medicine, Zhejiang University, Hangzhou, China; ^2^ Biomedical Informatics Research Lab, School of Basic Medicine and Clinical Pharmacy, China Pharmaceutical University, Nanjing, China; ^3^ Cancer Genomics Research Center, School of Basic Medicine and Clinical Pharmacy, China Pharmaceutical University, Nanjing, China; ^4^ Big Data Research Institute, China Pharmaceutical University, Nanjing, China

**Keywords:** breast cancer, subtyping, clustering analysis, transcriptomics, immune signatures, cancer immunotherapy

In the original article, there was a mistake in the **Legends** for “Figures 3–8” as published. The correct **Legends** appear below.

“FIGURE 3 | Comparisons of clinical features among the BC subtypes. **(A)** Comparisons of overall survival (OS) and disease-free survival (DFS) time among the BC subtypes by Kaplan–Meier curves. The log-rank test *p* values are shown. Comparisons of the proportion of high-grade (G3) tumors, the proportion of late-stage (stage III–IV) tumors **(B)**, and proportions of HER2+, TNBC, HR + tumors **(C)** among the BC subtypes in METABRIC. The Fisher’s exact test *p* values are shown.”

“FIGURE 4 | Comparisons of genomic features among the BC subtypes in TCGA-BRCA. Comparisons of TMB and neoantigen load **(A)**, SCNA scores **(B)**, and global methylation levels **(C)** among the BC subtypes. The one-tailed Mann–Whitney *U* test *p* values are shown in **(A,B,C)**. **(D)** Prediction of the scores (high (>median) versus low (<median)) of three immune signatures (NK cells, CD8^+^ T cells, and immune cytolytic activity) using TMB and SCNA score by the logistic regression model. TMB: tumor mutation burden. SCNA: somatic copy number alteration.”

“FIGURE 5 | Pathways upregulated in the BC subtypes. **(A)** The KEGG pathways upregulated in BC-ImH versus BC-ImL identified in the five BC datasets in common. **(B)** Spearman correlations between the enrichment scores of pathways upregulated in BC-ImH and immune scores in the five BC datasets. The immune score of a tumor represents its immune infiltration level, which was calculated by ESTIMATE (**Yoshihara, K., et al., 2013**).”

“FIGURE 6 | Comparisons of somatic mutation profiles among the BC subtypes. **(A)** Nine genes showing significantly different mutation frequencies among the BC subtypes in TCGA-BRCA. **(B)** Three genes show significantly different mutation frequencies among the BC subtypes in METABRIC. The Fisher’s exact test *p* values are shown.”

“FIGURE 7 | Heatmap showing differentially expressed proteins among the BC subtypes in TCGA-BRCA.”

“FIGURE 8 | Validation of the BC subtyping method in a single-cell RNA-seq dataset. **(A)** Hierarchical clustering of 317 tumor cells from ten BC patients based on the enrichment scores of four immune-related pathways. **(B)** Comparisons of the expression levels of 19 human leukocyte antigen (HLA) genes among the subtypes. One-way analysis of variance (ANOVA) test *p* values are shown. **(C)** Comparisons of proportions of TNBC, HER2+, and ER + tumor cells among the subtypes. The Fisher’s exact test *p* values are shown.”

The authors apologize for this error and state that this does not change the scientific conclusions of the article in any way. The original article has been updated.

